# Reliability, Validity, and Significance of Assessment of Sense of Contribution in the Workplace

**DOI:** 10.3390/ijerph110201594

**Published:** 2014-01-29

**Authors:** Jiro Takaki, Toshiyo Taniguchi, Yasuhito Fujii

**Affiliations:** 1 Department of Public Health and Occupational Medicine, Graduate School of Medicine, Mie University, 2-174 Edobashi, Tsu, Mie 514-8507, Japan; 2 Department of Welfare System and Health Science, Okayama Prefectural University, 111 Kuboki Soja, Okayama 719-1197, Japan; E-Mails: taniguti@fhw.oka-pu.ac.jp (T.T.); fujii@fhw.oka-pu.ac.jp (Y.F.)

**Keywords:** psychological distress, reliability, scale development, sense of contribution, sleep disturbance, validity

## Abstract

The purpose of this study was to assess the validity and reliability of the Sense of Contribution Scale (SCS), a newly developed, 7-item questionnaire used to measure sense of contribution in the workplace. Workers at 272 organizations answered questionnaires that included the SCS. Because of non-participation or missing data, the number of subjects included in the analyses for internal consistency and validity varied from 1,675 to 2,462 (response rates 54.6%–80.2%). Fifty-four workers were included in the analysis of test–retest reliability (response rate, 77.1%). The SCS showed high internal consistency (Cronbach’s α coefficients in men and women were 0.85 and 0.86, respectively) and test–retest reliability (intraclass correlation coefficient = 0.91). Significant (*p* < 0.001), positive, moderate correlations were found between the SCS score and scores for organization-based self-esteem and work engagement in both genders, which support the SCS’s convergent and discriminant validity. The criterion validity of the SCS was supported by the finding that in both genders, the SCS scores were significantly (*p* < 0.05) and inversely associated with psychological distress and sleep disturbance in crude and in multivariable analyses that adjusted for demographics, organization-based self-esteem, work engagement, effort–reward ratio, workplace bullying, and procedural and interactional justice. The SCS is a psychometrically satisfactory measure of sense of contribution in the workplace. The SCS provides a new and useful instrument to measure sense of contribution, which is independently associated with mental health in workers, for studies in organizational science, occupational health psychology and occupational medicine.

## 1. Introduction

Alfred Adler recognized the importance of *Gemeinschaftsgefühl*, commonly translated into English as “social interest”, which involves a desire to be a socially useful person. In Adler’s psychology, social interest is the main criterion for positive social adjustment, and one of the basic difficulties of people with psychological problems is that they lack such social interest. Therefore, the ultimate goal of Adlerian therapy is to enable a patient’s social interest. Ideally, in the Adlerian spirit, the person thereby becomes a true contributor to the betterment of society and one who feels at home in society [1].

Viktor Frankl developed logotherapy to help individuals find meaning in their life, which he believed is the primary motivating force in humans [2]. He suggested that one of the ways in which a person can find meaning in their life is by helping others [3].

Thus, both Adler and Frankl appear to have considered one’s sense of contribution as being important for mental health. For effective management in the workplace, Drucker insisted that the focus on contribution by itself supplies the basic requirement for successful human relations, including communication, teamwork, self-development, and development of others [4]. Such human relations can improve mental health.

Social capital can be defined as the norms of reciprocity and trust, formal and informal associations, and civic participation that facilitate collective action for mutual benefit [5]. Social capital has been found to be protective for mental health, and various underlying mechanisms have been proposed to explain the association between social capital and mental health, for example, social support, social network, and social control over health-risk behaviors [6]. However, these mechanisms have not been fully established [6]. Since a sense of contribution can be a consequence of social capital, we propose that sense of contribution may be one of these mechanisms.

The sense of making a contribution to others appears to play an important role in mental and physical well-being. Longitudinal studies have shown that older adults who felt that they were useless to others were more likely to have a disability or to die earlier than those who felt useful to others [7,8,9,10]. In the workplace, feeling useful to others has been found to be inversely associated with depression and sleep disturbances; with a worsening of the effort-reward balance, symptoms of depression increased, but feeling useful to others acted as a buffer [11]. Other research has found that a sense of contributing to society had a significant negative association with psychological distress among workers after adjusting for demographics, but the association did not remain significant after adjusting for job stress [12].

In the above studies, the participants’ sense of contribution was assessed by only a single question, such as “How often have you felt useful to your family and friends?” [7,11] or “Have you ever felt that your job contributed to society?” [12]. In general, multi-item measures are preferred to single-item measures because they provide increased information and statistical power. Moreover, the reliability of a questionnaire increases as the number of its items increases—especially below 18 items [13].

In this study, we developed a new scale of sense of contribution and assessed its reliability and validity. The study also examined two related concepts: organization-based self-esteem (OBSE) and work engagement. OBSE was defined as “the degree to which an individual believes him or herself to be capable, significant, and worthy as an organizational member” [14]. Work engagement refers to a positive, fulfilling, work-related state of mind [15]. Although these concepts are similar, they differ from a sense of contribution and were used to assess convergent and discriminant validity. We hypothesized that a sense of contribution has a moderate, but not excessively high, positive correlation with both OBSE and work engagement. With regard to criterion validity, we hypothesized that a sense of contribution is inversely related to poor mental health (e.g., psychological distress and sleep disturbance) based on Adler’s and Frankl’s theories [1,2,3].

## 2. Methods

### 2.1. Subjects

The subjects in this study were recruited from the workers (*n* = 3,070) at 272 health-care or welfare institutions in Japan. The questionnaires were mailed to the organizations and then distributed to the workers. The purpose and procedure of the survey were explained to the participants in the documents. Written informed consent was obtained from all participants. In all, 2,468 questionnaires were returned, which gave a temporal response rate of 80.4%. Listwise deletion was used in each analysis. Owing to missing data, the total number of men and women included in each analysis varied from 1,675 to 2,462 (final response rates varied from 54.6% to 80.2%). Among the participants, 54 workers at four healthcare or welfare institutions (response rate, 77.1%) answered the questionnaire twice with a 1-month interval for the analysis of test–retest reliability. This study was approved by the ethics committee of Okayama Prefectural University.

### 2.2. Measures

The self-administered questionnaire included demographic questions (age, gender, marital status, work shift, and work position), the new scale for sense of contribution, and measures of OBSE, work engagement, psychological distress, sleep disturbance, effort–reward imbalance, workplace bullying, and organizational justice. The “work shift” variable was defined as “shift work without night shift = 1; shift work with night shift = 2; regular daytime work = 3”. The “work position” variable was defined as “non-managerial worker = 1; managerial worker = 2”.

To determine the items that would constitute the new measure of sense of contribution—the Sense of Contribution Scale (SCS)—we examined questions used in previous studies about feeling useful to others or a sense of contribution to society [7,8,9,10,11,12] and selected from among them. Additionally, we also included new items and drafted seven items that were proposed to exhaustively and exclusively measure the sense of contribution. The SCS consists of the seven items that are measured on a four-point scale (1 = never; 2 = rarely; 3 = sometimes; 4 = frequently). The total score (ranging from 7 to 28) reflects one’s sense of contribution. Both English and Japanese versions of the SCS were prepared. The original items in Japanese were translated into English by two independent native English speakers and then back-translated into Japanese. The back-translation was then checked by the authors.

OBSE was measured with the OBSE scale developed by Pierce and his colleagues [14], which consists of 10 items. All items are scored on a five-point Likert-type scale, ranging from 1 (strongly disagree) to 5 (strongly agree). The OBSE scale has been translated into Japanese, and its eight-item version has been found to have acceptable reliability and construct validity [16]. Thus, we used the eight-item Japanese OBSE scale, whose total score ranges from 8 to 40. Higher scores indicate greater OBSE.

Work engagement was assessed using the short form of the Utrecht Work Engagement Scale (UWES) [15], which has been validated in Japan [17]. The UWES includes three subscales (each consisting of three items): vigor, dedication, and absorption. Vigor is characterized by high levels of energy, the willingness to invest effort in one’s work, and persistence in the face of difficulties. Dedication refers to being strongly involved in one’s work and experiencing a sense of significance, enthusiasm, inspiration, pride, and challenge. Absorption is characterized by being fully concentrated and engrossed in one’s work, whereby time passes quickly and there is difficulty in detaching oneself from one’s work. All items are scored on a seven-point Likert-type scale, ranging from 0 (never) to 6 (always). Each total score (ranging from 0 to 18) produces a score for each subscale. The total score of the three subscales (ranging from 0 to 54) is the score for work engagement.

The Kessler Psychological Distress Scale (K6), which consists of six items measured on a five-point scale (0–4), was used to evaluate psychological distress [18]. The total score (ranging from 0 to 24) has been used as a measure of psychological distress [18]. Higher scores indicate more severe psychological distress. The K6 has been translated into Japanese, and it has been shown to have acceptable internal reliability and validity for measuring DSM-IV mood and anxiety disorders, as assessed by diagnostic interviews administered by a lay interviewer in a sample community [19]. We used a cut-off point of >8 on the K6, which measures psychological distress in the Japanese population with 77.8% sensitivity and 86.4% specificity [20].

Sleep disturbance was assessed using the Pittsburgh Sleep Quality Index (PSQI), which is a self-administered questionnaire originally developed by Buysse and colleagues, that has been widely used for clinical and epidemiological research [21]. The PSQI assesses sleep quantitatively and qualitatively during the previous month and generates seven components (with scores of 0–3): sleep quality, sleep latency, sleep duration, habitual sleep efficiency, sleep disturbances, use of sleeping medication, and daytime dysfunction. The sum of these seven component scores produces one global score for sleep disturbance. Higher scores indicate higher sleep disturbance. The PSQI has been translated into Japanese, and its reliability and validity have been confirmed as excellent [22,23]. We used a cut-off point of >5 on the PSQI global score, which measures primary insomnia in the Japanese population with 85.7% sensitivity and 86.6% specificity [22].

Effort–reward imbalance was measured using the Effort–Reward Imbalance Questionnaire (ERIQ), developed by Siegrist [24]. The ERIQ has two main scales: extrinsic effort (six items; range 6–30) and reward (11 items; range 11–55), whose items are measured on a five-point scale. An effort–reward ratio, which is calculated as extrinsic effort divided by reward and multiplied by 6/11, has been used as an indicator of effort–reward imbalance; higher scores indicate more stressful situations [25]. The ERIQ has been translated into Japanese, and its reliability and validity have been reported as acceptable [26]. Effort–reward imbalance has been reported to be associated with poor mental health [27], and was used as a covariate in the analyses.

Workplace bullying was assessed using the Negative Acts Questionnaire-Revised (NAQ-R) [28]. The NAQ-R measures exposure to specific negative acts typical of bullying. It contains 22 items that refer to both direct and indirect behavior. Respondents indicate on a five-point scale (1 = never; 2 = now and then; 3 = monthly; 4 = weekly; and 5 = daily) whether they have experienced the designated negative acts in the context of their job. The sum of the answers to the 22 items produces a score for workplace bullying, in which higher scores indicate a greater amount of workplace bullying. The NAQ-R has been translated into Japanese, and its reliability and validity have been confirmed as acceptable [29]. Workplace bullying has been reported to be associated with poor mental health [30] and was used as a covariate in the analyses.

The Organizational Justice Questionnaire (OJQ) was used to assess organizational justice [31]. The OJQ consists of a seven-item scale assessing procedural justice and a six-item scale assessing interactional justice, both of which are measured on a five-point Likert-type scale, ranging from 1 (strongly disagree) to 5 (strongly agree). The former scale measures the degree of provision of relevant information to employees and the consistency of decision-making policy in the workplace; the latter scale measures the degree of fairness and consideration of respondents' supervisors. A total score for each OJQ subscale was calculated by averaging the item scores. The OJQ has been translated into Japanese, and its reliability and validity have been confirmed as acceptable [32]. Organizational justice has been reported to be associated with poor mental health [32] and was used as a covariate.

### 2.3. Statistical Analyses

Differences in continuous variables were compared between men and women using unpaired *t*-tests. Categorical variables were compared using chi-square tests. A gender difference was possible; therefore, we analyzed the data in men and women separately. An exploratory factor analysis was conducted on the SCS, in which factors with eigenvalues greater than 1 were extracted, using the least-squares method and Promax rotation to obtain the factor structure. Cronbach’s α coefficients were calculated for the SCS and other scales to test their internal consistencies. Because the PSQI global score and effort–reward ratio were not calculated as the sum of the answers to their items, Cronbach’s α coefficients were not calculated. Pearson’s correlations were used to assess the convergent and discriminant validity of the SCS. Criterion validity of the SCS was assessed by logistic regression that tested whether the SCS scores were negatively associated with psychological distress and sleep disturbance.

The test–retest reliability of the SCS score was determined using an intraclass correlation coefficient (ICC) [33]. Because of the small sample size, we analyzed it in men and women as a whole. The strength of agreement between test–retest responses for the continuous variable was characterized according to the ratings suggested by Robinson *et al.*: poor = 0–0.2, fair = 0.2–0.4, moderate = 0.4–0.6, substantial = 0.6–0.8, and almost perfect = 0.8–1.0 [34].

**Table 1:** Participant characteristics according to gender.

Characteristic	Men				Women				*p* ^a^
	*n*	%		Total *n* ^b^	*n*	%		Total *n* ^b^	
Married	263	61.0		431	1,181	58.1		2,031	0.271
Work shift				426				2,014	<0.001
Shift work without night shift	14	3.3			171	8.5			
Shift work with night shift	325	76.3			1,141	56.7			
Regular daytime work	87	20.4			702	34.9			
Managerial worker	76	18.0		423	273	13.9		1,968	0.030
	**α**	**Mean**	**SD**	***n*** **^b^**	**α**	**Mean**	**SD**	***n*** **^b^**	
Age (years)		33.3	7.3	432		41.2	12.0	2,021	<0.001
Sense of contribution (SCS)	0.85	18.5	3.8	424	0.86	19.0	3.6	1,996	0.004
Organization-based self-esteem (OBSES)	0.90	25.1	4.8	421	0.90	25.9	4.6	1,974	0.002
Work engagement (UWES)	0.95	25.3	12.3	428	0.94	27.4	12.4	1,985	0.002
Vigor	0.90	7.8	4.4	429	0.88	8.7	4.5	2,010	<0.001
Dedication	0.83	9.8	4.1	430	0.85	10.6	4.2	1,998	<0.001
Absorption	0.91	7.7	4.6	429	0.88	8.2	4.6	2,013	0.022
Psychological distress (K6)	0.92	12.4	5.9	415	0.92	11.6	5.6	1,967	0.016
Sleep disturbance (PSQI)		5.5	3.2	390		5.6	3.0	1,730	0.423
Effort (ERIQ)	0.84	17.5	5.1	415	0.86	16.5	5.3	1,920	<0.001
Reward (ERIQ)	0.79	41.1	6.9	397	0.80	42.8	6.6	1,772	<0.001
Effort–reward ratio (ERIQ)		0.83	0.40	395		0.75	0.35	1,739	<0.001
Workplace bullying (NAQ-R)	0.95	28.6	10.6	402	0.95	26.9	9.0	1,896	0.004
Procedural justice (OJQ)	0.90	3.2	0.8	421	0.93	3.2	0.8	1,940	0.526
Interactional justice (OJQ)	0.95	3.4	0.9	427	0.96	3.3	1.0	2,005	0.020

α = Cronbach’s α coefficient, SD = standard deviation, SCS = Sense of Contribution Scale, OBSES = Organization-based Self-esteem Scale, UWES = Utrecht Work Engagement Scale, K6 = Kessler Psychological Distress Scale, PSQI = Pittsburgh Sleep Quality Index, ERIQ = Effort–Reward Imbalance Questionnaire, NAQ-R = Negative Acts Questionnaire-Revised, OJQ = Organizational Justice Questionnaire; ^a^ Differences in continuous variables were compared using unpaired *t*-tests. Categorical variables were compared using chi-square tests; ^b^ *n* varies due to missing data.

All the *p* values were two-tailed, and *p* < 0.05 was taken as the threshold for significance. All statistical analyses were performed using SPSS version 20 (IBM Japan, Chuo-ku, Tokyo, Japan).

## 3. Results

The participants’ characteristics according to gender are shown in Table 1. Work shift, work position, age, the SCS score, OBSE, work engagement and its subscale scores, psychological distress, effort, reward, effort–reward ratio, workplace bullying, and interactional justice were significantly different between genders. Cronbach’s α coefficients for the SCS in men and women were 0.85 and 0.86, respectively. Cronbach’s α coefficients for other scales were >0.8 except for the reward score in men (0.790) and women (0.796).

The items of the SCS are shown in Table 2. In both genders, the exploratory factor analyses of the SCS obtained a one-factor structure.

The convergent validity of the SCS was supported by the findings that it had moderately positive correlations (*p* < 0.001) with OBSE, work engagement, and its three subscales (vigor, dedication, and absorption) in both genders, as we hypothesized (Table 3). Because the correlations were not excessively high (e.g., >0.70), the results also indicated the SCS has discriminant validity [35].

The criterion validity of the SCS is shown in Table 4. Changes in the odds ratios for psychological distress and sleep disturbance associated with a one-standard-deviation decrease in the SCS score showed that in both genders, the SCS scores were significantly and inversely related to psychological distress and sleep disturbance. Significant associations were found both in crude and in multivariable analyses that controlled for the covariates: that is, age, marital status, work shift, work position, OBSE, work engagement, effort–reward ratio, workplace bullying, procedural justice, and interactional justice.

The test–retest reliability of the SCS score was almost perfect (ICC = 0.91; 95% confidence interval, 0.85 to 0.95).

**Table 2:** Exploratory factor analysis of items of the Sense of Contribution Scale using a least squares method and Promax rotation.

Items	Factor loading	
	Men	Women
	*n* = 424	*n* = 1,996
1. I feel useful to my family.	0.50	0.46
2. I feel useful to my coworkers or subordinates in my workplace.	0.82	0.81
3. I feel useful to my superiors in my workplace.	0.85	0.81
4. I feel useful to my workplace partners or clients.	0.70	0.78
5. I feel useful to my friends who are not related to my work.	0.50	0.50
6. I feel useful for the continuance or growth of my workplace.	0.81	0.80
7. I feel useful to the community or the society.	0.59	0.65
Variance explained (%)	48.7	49.1

**Table 3:** Pearson’s correlation coefficients (*r*s) with sense of contribution.

Concept	Men		Women	
	*r*	*n*	*r*	*n*
Organization-based self-esteem	0.61	416	0.59	1,948
Work engagement	0.43	421	0.45	1,955
Vigor	0.42	422	0.41	1,979
Dedication	0.40	423	0.44	1,968
Absorption	0.38	422	0.39	1,982

All *p* values < 0.001.

**Table 4:** Associations of sense of contribution with poor mental health.

Dependent Variable	Men			Women		
	OR ^a^	95% CI	*n* ^b^	OR	95% CI	*n* ^b^
**Psychological distress**						
Crude	1.81	(1.43, 2.29)	409	1.60	(1.45, 1.77)	1,937
Adjusted for covariates ^c^	1.43	(1.02, 2.02)	350	1.28	(1.09, 1.51)	1,514
**Sleep disturbance**						
Crude	1.44	(1.17, 1.76)	385	1.36	(1.23, 1.51)	1,709
Adjusted for covariates ^c^	1.37	(1.01, 1.86)	325	1.37	(1.17, 1.60)	1,350

OR = odds ratio, CI = confidence interval; ^a^ Changes in the odds ratio associated with a one-standard-deviation decrease in the Sense of Contribution Scale score. All *p* values < 0.05; ^b^ *n* may vary due to missing data; ^c^ Age, marital status, work shift, work position, organization-based self-esteem, work engagement, effort–reward ratio, workplace bullying, procedural justice, and interactional justice.

## 4. Discussion

The present study investigated the validity and reliability of the SCS, a newly developed, seven-item questionnaire for sense of contribution, in the workplace. The SCS showed high internal consistency and test–retest reliability. Correlations between the SCS scores and the OBSE and work engagement scores were in the theoretically expected magnitudes and directions in both genders, providing support for the SCS’s convergent and discriminant validity. The criterion validity of the SCS was supported by its significant, inverse association with psychological distress and sleep disturbance in logistic regression models, independent of demographics and various psychosocial factors at work in both genders.

As mentioned earlier, previous research failed to find an association between a sense of contribution to society and psychological distress, after adjustment for job stress factors [12]. The failure to find an association could have been because a sense of contribution was measured by only one question in the study.

The strength of this study was that we used relatively large samples of workers to obtain reliable results. However, we must also note several limitations. First, all the measurements were self-reported, so, more objective measurements are needed in future studies. Second, the use of a cross-sectional design did not allow us to determine causality in our results, and future experimental intervention research is needed. Third, because we used convenience sampling, the results may not be applicable to the entire workforce. However, since we included workers from 272 organizations and obtained response rates ranging from 54.6% to 80.2%, some degree of generalizability can be expected.

Despite the above limitations, this study shows that the SCS is a psychometrically adequate measure. The SCS provides a new and useful instrument to measure a sense of contribution, which is independently associated with mental health in workers, for studies in organizational science, occupational health psychology, and occupational medicine.

## 5. Conclusions

The SCS is a psychometrically satisfactory measure of an individuals’ sense of contribution in their workplaces. The SCS provides a new and useful instrument to measure a sense of contribution, which is independently associated with mental health in workers, for studies in organizational science, occupational health psychology, and occupational medicine.
